# Metabolic Shift and Hyperosmolarity Underlie Age-Related Macular Degeneration

**DOI:** 10.3390/life14091189

**Published:** 2024-09-20

**Authors:** Laurent Schwartz, Jules Schwartz, Marc Henry, Ashraf Bakkar

**Affiliations:** 1 Assistance Publique des Hôpitaux de Paris, 75610 Paris, France; schwartzjules99@gmail.com; 2 Institut Le Bel, Université Louis Pasteur, 67070 Strasbourg, France; henry@unistra.fr; 3 Faculty of Biotechnology, October University for Modern Sciences and Arts, Giza 12451, Egypt; abakkar@msa.edu.eg

**Keywords:** macular degeneration, osmolarity, apoptosis, mitochondria, lipoic acid, methylene blue

## Abstract

Age-related macular degeneration (AMD) is both a poorly understood and devastating disease. Here, we analyze the physico-chemical forces at stake, including osmolarity, redox shift, and pressure due to inflammation. Hyperosmolarity plays a key role in diseases of the anterior segment of the eye such as glaucoma, cataracts or dry eyes, and corneal ulceration. However, its role in macular degeneration has been largely overlooked. Hyperosmolarity is responsible for metabolic shifts such as aerobic glycolysis which increases lactate secretion by Muller cells. Increased osmolarity will also cause neoangiogenesis and cell death. Because of its unique energetic demands, the macula is very sensitive to metabolic shifts. As a proof of concept, subretinal injection of drugs increasing hyperosmolarity such as polyethylene glycol causes neoangiogenesis and drusen-like structures in rodents. The link between AMD and hyperosmolarity is reinforced by the fact that treatments aiming to restore mitochondrial activity, such as lipoic acid and/or methylene blue, have been experimentally shown to be effective. We suggest that metabolic shift, inflammation, and hyperosmolarity are hallmarks in the pathogenesis and treatment of AMD.

## 1. Introduction

Age-related macular degeneration (AMD) affects roughly 20 million people in the United States and 196 million worldwide. AMD is a primary cause of severe vision impairment in the elderly, with an estimated 288 million people predicted to be affected globally by 2040 [1].

AMD is a poorly understood disease affecting mostly the elderly, resulting in visual loss. Usually, the peripheral retina is not affected but rather the macula and thus the ability to read [2,3,4]. Its course evolves with aging. In the beginning, vision is not affected but deposits in the retina (drusen) are formed. Drusen is a clinically noticeable localized yellow deposit of extracellular, polymorphous material at the interface between the RPE and the inner collagenous zone of Bruch’s membrane. It is usually bilateral but one eye can be affected before the second. The AMD is divided into two different groups. The first one is dry AMD where no neoangiogenesis can be seen. The second is the more aggressive wet AMD with choroidal neo-vessels. This abnormal growth of vessels from the choroidal vasculature to the neurosensory retina through Bruch’s membrane is responsible for hematoma and destruction of the macula. They can be occult and best seen via fluorescein angiography [5].

Hyperosmolality of the posterior segment causes metabolic shifts which in turn are responsible for aerobic glycolysis with increased lactate secretion by the Muller glial cells, which will feed the retinal cells. Hyperosmolarity also leads to neoangiogenesis [6,7]. The retina cannot metabolize this excess lactate, resulting in acidic intracellular pH, synaptic dysfunction, extracellular deposition (drusen), and ultimately apoptosis [8]. Therefore, we suggest that AMD can be viewed as a mitochondrial failure secondary to inflammation and metabolic disease.

## 2. Inflammation Is the Driving Force in AMD

A small proportion of AMD cases appear to have a genetic component [9]. These genetic features are rare in contrast to the most common sporadic ones. The vast majority of cases of AMD are sporadic. AMD, like cancer and Alzheimer’s disease, is strongly age-related [10].

Elderly people express low-level systemic inflammation [11]. Systemic inflammation plays a key role in the diseases of aging [11]. Inflammation, as seen in the process of aging, is a determinant in most of the diseases of the elderly [12]. The role of inflammation in AMD has been partially overlooked and is characterized by complement activation [13]. There are scanty reports about its importance but no clear explanation about its central role in this disease [14]. Another clue to the link between AD and AMD is the presence of beta-amyloid in the drusen [15].

Activation of inflammasomes in AMD patients leads to retinal neovascularization [16]. C-reactive protein, located in drusen in the choroid basal layer, regulates activated platelets and monocytes, participates in inflammatory pathways, and destroys choroidal cells [17,18].

Splenic monocytes play a role in chorioretinal infiltration, and suppression with angiotensin II receptor (ATR1) antagonists and splenectomy lowers subretinal mononuclear phagocyte accumulation and abnormal choroidal neovascularization development. In aged AMD-risk Apolipoprotein E-2 (ApoE2)-expressing mice, a chronic AMD model, ATR1 blockers and splenectomy also reduce chronic retinal inflammation and concomitant cone degeneration in these mice. Higher plasma angiotensin II levels were determined in AMD, supporting the key role of inflammation [19].

Enhanced retinal degeneration and cytokine response in a mouse model of dry age-related macular degeneration (AMD) have been demonstrated in the context of systemic inflammation from rheumatoid arthritis (RA). This study looked at the effect of Histone deacetylase (HDAC) inhibition on AMD progression in the presence of systemic inflammation [20]. HDAC inhibition minimized the cumulative effect of NaIO3-induced retinal degeneration in the presence of systemic inflammation caused by collagen-induced arthritis (CIA), as evaluated by optical coherence tomography (OCT). Furthermore, HDAC inhibition in CIA + sodium iodate (NaIO3)-treated mice lowered cytokine production. These findings indicated the therapeutic potential of HDAC inhibitors for dry AMD therapy [20].

## 3. Increased Osmolality as the Driving Force for AMD

Inflammation is a clinical feature that can be caused by factors as diverse as heat, freezing temperature, trauma, or multiple chemicals resulting in vascular leakage [21,22]. Inflammation (a clinical feature) is closely related, if not synonymous, to hyperosmolality (a physical feature) [23,24].

Osmotic pressure was discovered by Jean Antoine Nollet in 1748. Its role has been mostly studied in physiology. Blood pressure allows water and small molecules such as glucose to be extruded outside the capillaries and feed the cells of the body. In healthy tissues, macromolecules (such as large proteins) cannot flow outside the capillaries, thus creating an osmotic gradient. This gradient is responsible for the return of water to the venous circulation. Similarly, in the kidney, most proteins cannot pass into the filtrate, resulting in water’s movement out of the capsule towards the glomerulus. Osmolarity is responsible for the return of most water toward blood circulation [25].

The role of osmotic pressure is not limited to normal physiology but also plays an essential role in many diseases. There is little—if any—protein in the extracellular fluid. The osmolarity is around 300 mOsm/L. Most proteins are confined in the vascular space where the osmolarity is higher (330 mOsm/L). In the case of inflammation, there is always vascular leakage and the proteins are then present in the extracellular space. Animal models of inflammation demonstrate that in inflammatory fluids, whatever the cause, there is protein content, resulting in increased osmolarity. Increased osmolarity results in inflammation. Increased extracellular osmolarity stimulates cytokine synthesis, such as that of interleukins or TNF alpha, and results in the proliferation and activation of immune cells [23].

There is substantial evidence that supports the involvement of osmotic pressure/hyperosmolarity in AMD. A significant positive correlation was found between reduced plasma colloid osmotic pressure (COP) and an enhanced visual field in nonexudative or dry AMD. Therefore, COP plasma level modification may help AMD patients [26]. Hyperosmotic stress has also been shown to have several effects on RPE cells [27]. These effects include the accumulation of organic osmolyte in RPE cells [28], cytoskeleton rearrangement [29], cell cycle arrest, regulatory volume increase [30], and the alteration of RPE’s electrical characteristics [31].

It has been shown that intracellular organic osmolyte accumulation in RPE cells is a primary consequence of hyperosmolar stress. Hence, there is an increase in the intracellular sorbitol concentration, acting as an osmolyte to shield cells from hyperosmotic-induced cell shrinkage [27,32]. Rearrangement of the cytoskeleton is another effect of hyperosmolar stress on RPE cells. Hyperosmotic stress applied to the cultivated rabbit RPE cells increased the expression of lysyl oxidase, an extracellular amine oxidase directing the maturation of collagen and elastin. The cohesive force between RPE and Bruch’s membrane may arise from their interaction, and the lysyl oxidase generated in RPE cells may play a role in this interaction [27,29]. Moreover, hyperosmolar stress led to cell cycle arrest in human RPE-derived cell line ARPE-19 via a subset of regulated genes identified by gene expression profiling in these cells [8,27]. Furthermore, cell volume regulation is another outcome of hyperosmolar stress on RPEs. Regulatory volume increase (RVI) was brought about by the hyperosmolar-induced shrinkage of human and frog RPE [30,33]. Additionally, hyperosmolar stress reduced transepithelial electrical resistance (TER) from the bovine RPE when applied to the apical side of the epithelia [27,31]. On the other hand, several other effects have been documented regarding angiogenic growth factors. Hyperosmolarity stimulates basic fibroblast growth factor (bFGF) and heparin-binding EGF-like growth factor (HB-EGF) gene transcription and bFGF secretion from RPE cells. It has been suggested that a high dietary salt intake that causes osmotic stress may exacerbate neovascular retinal disorders by stimulating the synthesis of angiogenic factors in RPE cells [7]. Indeed, it was suggested that excessive salt consumption in the diet, which raises extracellular osmolarity [34,35], may affect RPE cells directly and independently of hypertension. Among these effects are the upregulation of IL-1β expression and the gene expression of several angiogenic factors [7]. The generation of angiogenic factors triggered by salt could potentially accelerate the progression of choroidal neovascularization and edema, whereas IL-1β could potentially exacerbate the retinal inflammation linked to AMD [7]. Moreover, hyperosmolarity triggers the transcription of aquaporin-5 (AQP5), aquaporin-8 (AQP8), and vascular endothelial growth factor (VEGF) genes, causing RPE cells to secrete VEGF. A high-salt diet that causes osmotic stress may exacerbate neovascular retinal disorders and edema by inducing RPE to produce more VEGF. Furthermore, under hypoxic conditions, AQP5 downregulation may impede edema clearance [36].

## 4. Osmosis in the Posterior Segment

The role of osmolality has been demonstrated in the pathologies of the anterior segment of the eye, such as dry eye [37]. It has been shown that tear osmolarity is the most useful single parameter for diagnosing and classifying dry eye conditions [37]. Tear osmolarity outperformed the other six tests in terms of diagnostic accuracy. The most sensitive threshold between normal and mild or moderate patients was shown to be 308 mOsms/L, whereas the most specific was 315 mOsms/L for corneal ulceration [38]. It has been suggested that greater palpebral fissure width promotes tear film evaporation, increasing tear film osmolarity and causing ocular surface injury and glaucoma [39]. Cataracts can also be caused by hyperosmolality [40]. Glaucoma is known to be caused by local hyperosmolarity [41].

The posterior segment of the eye has one of the highest metabolic rates of the body, requiring a rich supply of oxygen and other nutrients [42]. While the central retinal artery provides the inner retina’s blood supply, most of its oxygen demand (seven times greater than that of the brain per mass unit) is supplied by diffusion from the underlying choroid, which is the sole supply of the avascular fovea. The choroid has the highest rate of blood flow per weight of any tissue [43]. The choroid, because of its intense blood flow and the permeability of the Bruch’s membrane (BM), has a major role in the thermoregulation of the macula. The innermost layer of the choroid is the Bruch’s membrane, a 2–4 μm thick elastic sheet. At a young age, it is permeable to liquids, ions, and small proteins [44].

There is evidence of a dialysis-like phenomenon in the eye. Like in an artificial kidney cartridge, where there is the concomitant circulation of arterial blood and dialysate, the choroidal and the retinal fluid circulation are separated by the Bruch’s membrane. The Bruch’s membrane is semi-permeable and this membrane is formed of glycated proteins such as heparin. The flow of the choroidal arteries is about 10 to 20 times higher than the flow of the retinal arteries [43,45], increasing the hydrostatic pressure in the retina.

## 5. Aging of the Posterior Segment: Vascular Changes

During aging, there is a clear decrease in the perfusion of the choroid. The normal thickness of the choroid, measured in younger emmetropes (<27 years old) by EDI-OCT, varies from 264 to 436 µ [46]. It decreases by 15.6 µm per decade [47,48]. The Bruch membrane thickens, further altering exchanges [44]. The choroidal blood flow decreases, as does the porosity of the Bruch membrane. The decreased perfusion will result in ischemia and, therefore, the impairment of retinal pigment epithelium (RPE) hemodynamic cellular antioxidant properties. The retinal pigment epithelium (RPE) is unable to adequately manage such increased oxidative stress either by the apical synthesis of reticular pseudo drusen (RPD), also named subretinal drusenoid deposits (SDDs), or by the more common basal exocytosis of multiple drusen composed of lipofuscin and bis retinoids [13].

The retinal pigment epithelium (RPE) separates the retinal (apical) and choroidal (basal) environments and contributes to the blood–retinal barrier (BRB), which provides a proper environment for photoreceptor cells. The osmolality on the choroidal side is higher than on the retinal side in physiological conditions [49]. Thus, the osmotic gradient from the apical to basal sides is thought to elevate transepithelial electric resistance (TER). TER reflects ion permeability across the epithelia [50]. So, gradients illustrate the direction of epithelial function or pathological changes according to apical and basal osmotic conditions through hydrostatic pressure (HP) [49]. The accumulation of these multiple deposits and wastes of various molecules will also result in a significant increase in osmolarity [23].

## 6. Increased Osmolarity and AMD

To the best of our knowledge, osmolarity has not been measured in any pathology of the posterior segment, but there is indirect and direct evidence of its importance in AMD. AMD is characterized by multiple features, such as infiltration of the retina by inflammatory cells, proliferation of fibroblasts, and new blood vessel formation. In the neovascular subfoveal membranes, the proliferation of fibroblasts results in the formation of a conjunctival tissue incorporating the new vessel network [51].

The secretion of vascular growth factors that leads to wet macular degeneration by the retinal human pigment cells is increased by osmolarity [7,36]. Retinal detachment can be induced by the intravitreal injection of a hyperosmotic solution [52]. Fibroblast proliferation could be a consequence of increased osmolarity [53].

The best demonstration of the key role of osmolarity in AMD comes from animal models [54]. The retina can be damaged by either blue light or a laser. This results in vascular damage and, in turn, increased osmolarity because of an extravascular protein leak. Polyethylene glycol (PEG) is a chemically inert but osmotically active chemical. PEG is not metabolized in vivo [55]. The subretinal injection of PEG results in choroidal neovascularization [55] and the formation of structures resembling drusen [56], resulting in the loss of photoreceptors. There is cell death and atrophy, as is seen in AMD. The formation of choroidal neovascularization (CNV) in PEG-treated mice indicates that PEG can be used to induce CNV. In vitro, human adult retinal pigment epithelial-19 (ARPE-19) cells were treated with various doses of PEG and experienced cell death. However, the authors failed to link PEG to the osmolarity. Similarly, the intravitreous injection of hyperosmolar mannitol results in serous retinal detachment [55,56].

## 7. Metabolic Shift Induced by Increased Pressure

To perform their normal physiological functions, cells must maintain an intracellular pH (pHi) within the physiological range. Intracellular enzyme activity, cytoskeleton component integration, and cellular growth and differentiation rates are all closely associated with pHi [57]. A fall in pHi decreases neuronal activity and is responsible for apoptosis [58].

In Alzheimer’s disease, there is a shift toward intracellular acidosis and apoptosis. It has been demonstrated that neurons feed on lactate secreted by glial cells [59]. In the case of AD, there is increased lactate secretion as measured by spinal fluid [60].

The increased secretion of lactate by glial cells results in increased uptake by neurons and intracellular acidosis [59]. This is the inverse of Warburg’s effect, as first described by [61]. The fall in pHi may result in neuronal cell death.

A similar scenario is probably at play in AMD. Neurons feed on lactate released by glial cells, and similarly, retinal cells feed on lactate released by Muller cells [62]. The latter, akin to glial cells in the brain, metabolize glucose to lactate, which is preferentially taken up by photoreceptors as a fuel for their oxidative metabolism [63]. Even in the presence of glucose and oxygen, cultured human Müller cells obtain most of their ATP from aerobic glycolysis and display a low rate of oxygen consumption [64]. They feed lactate to the retinal cells. Indeed, the increased secretion of lactate will have multiple consequences: increased urinary secretion of lactate [65] and increased secretion of markers of inflammation [66] and VEGF [67]. The acidic pH plays a crucial role in retinal cell death [68].

Inflammation increases lactate secretion, resulting in increased serum and urine levels of lactate [69,70]. It has been shown that in response to osmotic stress, cells display acute metabolic remodeling via the control of pyruvate dehydrogenase phosphorylation through direct osmosensing in mitochondria [71]. Table 1 summarizes the metabolic characteristics of AMD.

**Figure 1:**
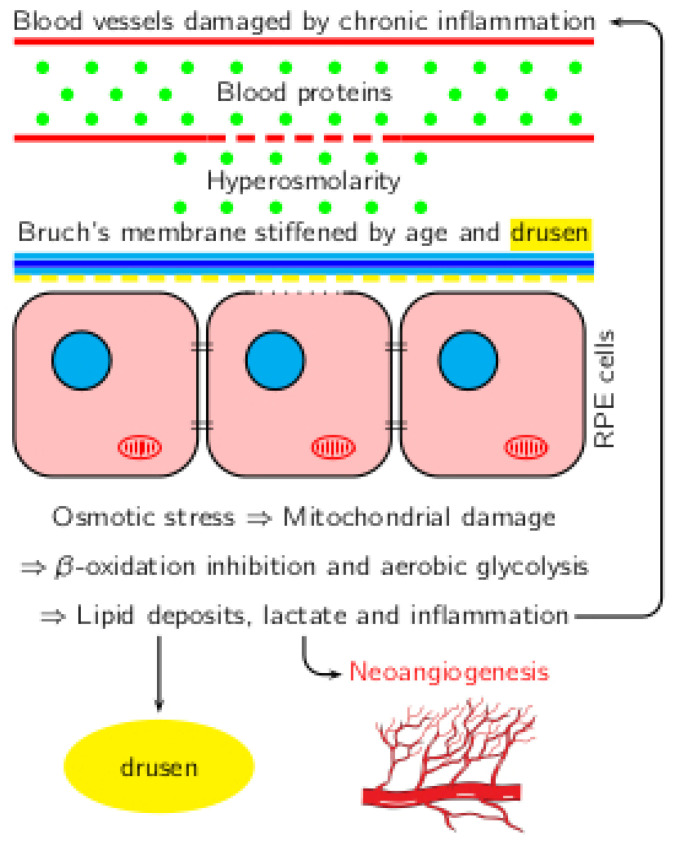
Correlation between inflammation, hyperosmolarity, and metabolic shift in AMD.

The correlations between inflammation, hyperosmolarity, and metabolic changes in AMD are shown in Figure 1.

## 8. Novel Therapeutic Approaches to AMD

There are very limited data on the metabolic pathways involved in AMD. The crucial enzymatic activities of most enzymes in glycolysis and mitochondria have not been measured. However, it has been recently shown that homocysteine causes a metabolic shift from mitochondrial respiration to a high rate of glycolysis in RPE cells in AMD [72]. In RPE cells treated with homocysteine, glycolysis was decreased by blocking N-methyl-D-aspartate receptors (NMDARs) or inhibiting GLUT-1. Consequently, NMDAR or glycolysis may be a novel treatment strategy for AMD [72].

Moreover, some authors targeted the oxidative stress induced in RPE cells [73]. The degeneration of RPE cells linked to the pathogenesis of AMD is mostly caused by oxidative stress. Age-related macular degeneration (AMD) is associated with the epithelial–mesenchymal transition (EMT) of retinal pigment epithelial (RPE) cells. It has been demonstrated that ERK is an important regulator of several NaIO3-induced signaling pathways that coordinate the epithelial–mesenchymal transition (EMT) program in RPE cells. The authors demonstrated that inhibiting ERK could be a potential therapeutic method for treating AMD [73].

Most treatments aim not at the root cause of AMD but at the symptomatic treatment of neo-vessel AMD with laser [74]. Other drugs inhibit the neoangiogenic process by intravitreal injection of drugs, such as Lucentis, which targets the growth factor VEGF [75]. However, resistance to anti-VEGF has been documented [69]. On the other hand, the treatment of dry AMD has remained elusive so far but multiple clinical trials are ongoing [76].

Some authors advocate a shift toward a ketogenic diet. The implementation of the ketogenic diet was based on the clinical observation that fasting had beneficial effects in the control of epileptic seizures [77]. A ketogenic nutritional approach is characterized by the consumption of foods with a high fat content and adequate protein amounts for growth but insufficient levels of carbohydrates for metabolic needs, thus forcing the body to primarily use fat as a fuel source for the eye [78]. To date, no clinical trials are studying the effect of a ketogenic diet on AMD patients, although investigations in mouse and zebrafish models have revealed potential neuroprotective roles of the ketogenic diet and/or its derivatives [79,80]. 

Ryals et al., 2020 [81] examined retinal degeneration in a mouse model (rd 10) of photoreceptor degeneration and demonstrated that only a combination of a ketogenic and low-protein diet produced beneficial outcomes. The rd10 mice kept on the combination diet had significantly increased electroretinogram (ERG) responses, as evidenced by a 1.8-fold increase in both b-wave phototropic response and a-wave scotopic response (indicative of rod photoreceptors and visual functioning during darkness). This combination diet resulted in a nearly two-fold increased photoreceptor layer, indicating improved photoreceptor function and survival [81]. These studies might provide support to the idea that a ketogenic diet may potentially help to preserve sight in AMD [79,80].

In AMD, supplementation with lipoic acid improves long-term vision [82], suggesting the key role of pyruvate dehydrogenase. A total of 100 dry AMD patients (60–83 years old) were randomly assigned to a lipoic acid (LA) treatment group (n = 50) or a placebo control group (n = 50). These results indicate that LA treatment improves vision-related quality of life in patients with dry AMD possibly by improving metabolic fluxes. Methylene blue, a century-old drug, can receive two electrons from NADH in the presence of complex I and donate them to cytochrome C, providing an alternative electron transfer pathway in defective mitochondria [83]. Methylene blue is an autoxidizable phenothiazine with potent oxidant and metabolic-enhancing properties. Methylene blue is reduced by accepting electrons from reduced electron transport donors and methylene blue transfers electrons to oxygen to form water, thus maintaining the activity of the electron transport chain [84]. Moreover, methylene blue decreases Warburg’s effect induced by increased osmolarity and lowers lactate secretion [85].

In animal models of AMD, methylene blue prevents neurodegeneration caused by rotenone in the retina [86] and prevents damage caused by acute ocular hypertension in rats [85].

## 9. Conclusions

This work strongly suggests that macular degeneration, like inflammation, is a straightforward consequence of increased osmolarity. In animals, drugs that induce hyperosmolarity cause AMD. Hyperosmolarity results in intracellular acidosis and apoptosis. Treatment should aim at normalizing the pressure and limiting the metabolic consequences of increased pressure. Drugs normalizing metabolic abnormalities in AMD such as methylene blue and α-lipoic acid appear to be promising. 

## Figures and Tables

**Table 1:** Comparison of metabolic characteristics of AMD.

Characteristic	Macular Degeneration
Extracellular osmolarity	Increases
Oxidative phosphorylation	Decreases in photoreceptors
Extracellular lactate concentration	Increases
Intracellular pH	Decreases in photoreceptors
Cell ecosystem	Photoreceptors + Müller cells
